# A Mixture of Free and Microencapsulated Essential Oils Combined with Turmeric and Tannin in the Diet of Dairy Cows: Effects on Productive Efficiency and Animal Health

**DOI:** 10.3390/ani15111588

**Published:** 2025-05-29

**Authors:** Emeline Pizzolatto de Mello, Miklos Maximiliano Bajay, Tainara Leticia dos Santos, Renato Santos de Jesus, Guilherme Luiz Deolindo, Luisa Nora, Mario Augusto Tortelli, Gilnei Bruno da Silva, Daiane Manica, Margarete Dulce Bagatini, Francisco Machado, Aleksandro S. da Silva

**Affiliations:** 1 Programa de Pós-Graduação em Zootecnia, Universidade do Estado de Santa Catarina (UDESC), Chapecó 89815-630, Brazil; emeline@unochapeco.edu.br (E.P.d.M.); miklos.bajay@udesc.br (M.M.B.); 07719519906@edu.udesc.br (T.L.d.S.); mario.torteli0900@edu.udesc.br (M.A.T.); 2 Departamento de Zootecnia, Universidade do Estado de Santa Catarina (UDESC), Chapecó 89815-630, Brazil; renato.jesus0026@edu.udesc.br (R.S.d.J.);; 3 Programa de Pós-Graduação Multicêntrico de Bioquímica e Biologia Molecular, Universidade do Estado de Santa Catarina (UDESC), Lages 88520-000, Brazil; guilhermeluizd@outlook.com (G.L.D.); luisa.nora22@gmail.com (L.N.); gilneibrunosilva@gmail.com (G.B.d.S.); 4 Centro de Ciências da Saúde, Universidade Federal Fronteira Sul, Chapecó 89815-899, Brazil; daianemanica2011@gmail.com (D.M.); margarete.bagatini@uffs.edu.br (M.D.B.)

**Keywords:** carvacrol, thymol, cinnamaldehyde, curcumin, polyphenols

## Abstract

This study aimed to evaluate whether the addition of a phytobiotic additive formulated based on cinnamon and oregano essential oils (50% free and 50% microencapsulated) combined with turmeric extract and tannins to the diet of cows has beneficial effects on health, productivity, and milk quality. In a completely randomized design, eighteen Jersey cows were used in a compost barn system over 45 days. The cows were divided into two homogeneous groups: one control (without additive; *n* = 9) and another treatment (with a phytobiotic at a dose of 2 g/cow/day; *n* = 9). The diet was formulated based on corn silage, hay and concentrate for daily 30 L/cow production. Blood and milk samples were collected at 15-day intervals. There was a treatment × day interaction: cows that consumed the phytobiotic additive produced a more significant amount of milk at days 14, 17, 18, 30, 39 and 45 (*p* ≤ 0.05). When we corrected milk production for fat percentage, we observed higher milk production in the cows that consumed phytobiotics compared to the control during the experimental period (*p* = 0.01). The feed intake of cows fed phytobiotics was lower (*p* = 0.01). Thus, feed efficiency was better in cows that consumed phytogenics. There was a higher percentage of fat in the milk of cows that consumed phytobiotics and a higher amount of polyunsaturated fatty acids compared to the control (*p* = 0.02). There was an increase in total protein and globulin levels (*p* = 0.01), which may be associated with the interaction of the antimicrobial, antioxidant, and immunomodulatory properties of the phytobiotic additive. An increase in immunoglobulins (*p* = 0.01) and a reduction in acute-phase proteins (*p* ≤ 0.05) were observed in the blood of cows in the phytobiotic group. Lower levels of TNF-α, IL-1β and IL-6 and higher levels of IL-10 in the serum of cows that consumed the phytoactive (*p* = 0.01) reaffirm the anti-inflammatory effect of the additive. Lower levels of lipid peroxidation (TBARS) and reactive oxygen species (ROS) were observed in the serum of cows in the phytobiotic group. Greater catalase and superoxide dismutase activity was observed in cows that consumed the phytogenic (*p* < 0.01). Therefore, it can be concluded that the additive in question has antioxidant, immunological, and anti-inflammatory actions and has the potential to improve productive performance when corrected for milk fat.

## Simple Summary

The increasing use of feed additives aims to increase productivity, but it can also improve cow health. In this study, the inclusion of a phytobiotic additive based on a combination of oregano and cinnamon essential oils (free and microencapsulated) associated with turmeric extract and tannin in the diet of Jersey cows increased milk production and was also capable of increasing milk fat, with an emphasis on a higher proportion of polyunsaturated fatty acids and unsaturated/saturated ratio. The ingestion of the phytoactive by the cows had an anti-inflammatory effect, capable of reducing pro-inflammatory markers, such as acute-phase proteins, but stimulated the production of immunoglobulins. Lower levels of lipid peroxidation and reactive oxygen species indicate that the additive has an antioxidant effect and can reduce and minimize physiological oxidative stress in lactating cows.

## 1. Introduction

Maintaining animal health has become a priority among producers in intensive production systems [1]. The aim is to improve the condition of animals against metabolic diseases, oxidative stress, and immunological challenges that may compromise growth performance as well as the longevity of dairy cows in the face of problems that often require veterinary interventions and the use of anti-inflammatory drugs and antibiotics, which increase production costs, in addition to the likelihood of leading to the development of antimicrobial resistance, a global public health concern. Therefore, the search for alternative and sustainable solutions has gained prominence; among them, phytobiotic additives and compounds of plant origin with functional properties have shown promise [2]. Essential oils (EOs) are recognized as effective natural additives that modulate ruminal fermentation, mitigate methane gas production, increase nutrient digestibility, and contribute to improved production efficiency and ruminant health [2,3]. The microencapsulation of EOs is a technological method that protects bioactive compounds and optimizes their release in the digestive tract of animals, increasing their effectiveness when the objective is absorption [4]. In addition, EOs have antimicrobial and antioxidant actions, modulate ruminal microbiota, and promote greater fermentation efficiency [5].

Curcumin, derived from Curcuma longa, is widely recognized for its antioxidant and anti-inflammatory properties, which contribute to reducing oxidative stress and improving liver function in cattle [6]. Tannins, in turn, protect dietary proteins against ruminal degradation, improve feed efficiency, and contribute to the reduction in methane emissions, which makes this synergistic interaction potentially beneficial to animal health and makes the nutrients present in the diet more efficient, in addition to ensuring animal welfare [7]. In this study, we used a combination of cinnamon and oregano EOs in their free and microencapsulated form associated with turmeric extract and tannin, which, in a previous survey of the group, enhanced the weight gain of cattle in confinement [8], unlike another study of ours with a similar additive, the only difference being that the EOs were only in microencapsulated form, with initial positive effects on weight gain that were not maintained throughout the experimental period in the rearing phase [9]. In two studies [8,9], an anti-inflammatory and antioxidant effect was observed, in addition to a hepatoprotective effect, which, according to the authors, may is responsible for enhancing the growth performance of beef cattle. Our hypothesis is that the anti-inflammatory, antioxidant and antimicrobial effects of the components present in the additive will enhance productivity since these known effects of phytogenic improve animal health. Therefore, the aim of this study was to evaluate whether the addition of a mixture of EOs (free and microencapsulated), combined with turmeric extract and tannin, to the diet of lactating cows had beneficial effects on animal health and metabolism, modulated the immune system, and stimulated the antioxidant response, resulting in improved growth performance of Jersey cows at peak production.

## 2. Materials and Methods

### 2.1. Additive

The phytobiotic blend was the Phyto Rumen Plus^®^ (Tecphy, Canelinha, Brazil), a trade name for The phytogenic blend contains in its formulation 1/3 of the phytogenic mixture corresponding to the microencapsulated EO of cinnamon (cinnamaldehyde) and oregano (carvacrol and tymol) (a ratio of 50:50), 1/3 non-encapsulated EO of cinnamon and oregano (50:50%) and 1/3 of turmeric extract (4.5–5% curcumin), and tannin. The blend contained 166 mg per concentrate [8], resulting in an average daily intake of 2 g of animal phytogenic compounds. The dose was defined based on previous studies by our research group and respected the commercial indication of the product.

### 2.2. Characterization of Experimental Environment

The experiment was carried out in the ruminant sector of the Experimental Farm of the Centro de Educação Superior do Oeste (FECEO) of the Universidade do Estado de Santa Catarina (UDESC), located in the city of Guatambu/SC, Brazil. In a compost, barn-type shed with an area of 384 m^2^, with a wood shaving/sawdust bedding area (15.2 m^2^ per cow), a feeding track with individual feeders was used, where each canister contained a cow. A robotic milking system with free access to cows was used in this study, configured to allow up to four milkings per cow per day. In addition, the facility had available smart feeders (Intergado^®^, Betim, Brazil) that identified cows and quantified their consumption during the night.

### 2.3. Animals, Experimental Design and Diet

**Figure 1:**
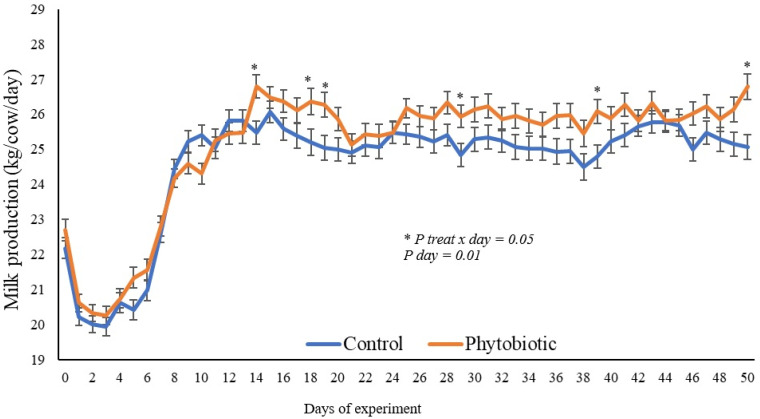
The daily milk production of cows fed phytobiotics compared to cows in the control group. * indicates the difference between groups owing to the treatment × day interaction.

The experiment involved 18 multiparous Jersey cows, between 21 and 30 days of lactation, divided into two groups of 9 cows each, according to the average milk production in the last 7 days before the experiment (day 1). Prior to the experiment, postpartum cows consumed a diet (corn silage and concentrate) with a low amount of starch in the feeders twice a day and had access to millet pasture, which explains why milk production on day 1 was on average 22.5 kg, reducing in the first seven days (Figure 1), and when there was a change in diet, there was a reduction in consumption (animals were removed from the pasture); feeding was only in the feeders, as described below.

The phytobiotic group received the concentrate with the addition of 2 g/day of the phytobiotic (Phyto Rumen Plus^®^, Tecphy, Canelinha, Brazil) via the concentrate in the individual feeder during the two feedings in the morning and afternoon, and the control group received the basal concentrate without the inclusion of the additive. It is important to emphasize that we chose to add additives only to the feedings with controlled quantities to ensure the total consumption of the additive at the scheduled dose of 2 g/cow/day. As there was no leftover feed during the entire experimental period, we can guarantee that 100% of the additive was consumed.

The diets were formulated according to the nutritional requirements of the NRC [10] for daily 30 L/cow production. The feed was formulated based on corn silage (44.74%), Tifton 85 hay (7.03%), and concentrate (48.23%) provided to the cows in the form of a basal diet (20 kg dry matter intake (DMI), which corresponded to a calculated composition of 16.9% of crude protein/kg DM, and net energy lactation (NEL) of 1.59 Mcal/kg DM (Table 1). During the day, feeding was performed in individual feeders (animals were trapped by a cage for 90 min), with access to water and drinkers between feeders, twice a day (07:00 and 16:00). At night, the cows had access to smart feeders that provided the daily feed intake for each cow (Intergado^®^, Ponta Agro, Betim, Brazil), which contained the total diet using the concentrate without additives, as previously mentioned. Water was provided ad libitum, as the cows had access to automatic drinkers spread throughout the facility.

A robotic milking system with a free-flow system was used for the cows to be milked; as an attraction for seeking milking, the cows received pelleted feed at each visit, limited to 3 kg/day. Therefore, the following was considered when measuring the dry matter intake (DMI) of the cows: diet in individual feeders (during the day) + diet in automatic feeders (during the night) + robotic concentrate (during milking).

### 2.4. Data and Sample Collection

The data on the daily feed intake were obtained using a robotic milking system (pelletized feed), an innovative feeder system, and by weighing the leftovers in the individual feeders. Milk production was performed daily using the milking system, allowing the verification of teat production and total milk production. Based on this information, the productive efficiency of the cows was calculated. Milk production corrected for 4% fat (4% FCM) was estimated using the equation proposed by the NRC (2001) [10]: 4% FCM = 0.4 × (kg of milk produced) + 15 × (% fat) × (kg of milk produced).

Blood was collected during the 45 days of the experiment at 15-day intervals, always in the morning (between 7:30 AM and 9:30 AM), before feeding the individual feeders. On the day of collection, all animals were apparently healthy, and no cow was apparently in estrus.

Samples were collected on days 14, 28 and 45 for composition and quality analysis. Milk collection was performed using a robotic milking system, which allows the collection of a homogeneous sample of the entire lactation period of the cow, with the material allocated in tubes (50 mL) containing preservatives (bronopol). The collected material was sent on the same day to a specialized commercial laboratory authorized by the Brazilian federal government.

Milk (50 mL) was collected on day 45 to determine the fatty acid profile. Blood samples were collected in tubes with a clot activator (4 mL) to separate blood serum and in tubes with 10% EDTA anticoagulant (4 mL) for blood count analysis and tubes with sodium citrate for the analysis of antioxidant enzymes in whole blood. After collection, the material was stored in a thermal box with ice (temperature ± 10 °C) until processing within 3 h. The tubes with the clot activator were centrifuged (820× *g* for 10 min), and the serum was collected and stored in microtubes at −20 °C.

Two hundred grams of samples of a partially mixed ration (PMR) supplied in individual and automatic feeders were collected throughout the experiment (days 1, 10, 20, 30 and 45), totaling 1 kg of the PMR that was frozen (−20 °C) until analysis. This material, as well as the robot feed (supplied during milking) collected on the same date as the PMR, were subjected to physical–chemical analyses described below.

### 2.5. Laboratory Analysis

#### 2.5.1. Chemical Composition of Feed

Initially, the feed samples were partially dried in a forced ventilation oven at 54 °C for 72 h, after which each sample was ground to pass through a 0.5 mm screen using a mill grinder (Retsch zm 200 (Retsch, Haan, Germany), ring sieve size: 0.75 mm) before chemical analysis. For dry matter (DM) evaluation, samples were placed in a forced ventilation oven at 105 °C for 24 h and then weighed. Crude protein quantification was performed using the Kjeldahl method (Method 2001.11), which includes the digestion, distillation, and titration phases [11]. The ash content, which indicates the mineral matter in the samples, was measured by incinerating the samples in a muffle furnace at 600 °C for six hours, eliminating the organic matter and retaining only the ashes, which were weighed to determine the percentage calculation. The ether extract was quantified according to the manufacturer’s recommendations using an automatic fat and lipid extractor (SER 158/6 Velp Scientifica^®^; Usmate Velate, Italy). Finally, neutral detergent fiber (NDF) analysis was performed according to the methods described by researchers [11,12].

#### 2.5.2. Hemogram

Hematological variables were obtained from blood samples collected in tubes with EDTA and analyzed using the automatic hematological analyzer VET3000^®^ (EQUIP, Itatiba, Brazil). This analyzer determines the counts of leukocytes, lymphocytes, granulocytes, monocytes, erythrocytes, and platelets, as well as the concentration of hemoglobin (g/dL) and hematocrit (%).

#### 2.5.3. Serum Biochemistry

Blood samples collected in tubes without anticoagulant were centrifuged in a QUIMIS (São Paulo, Brazil) tube centrifuge for 10 min at 820 g to obtain blood serum. The serum was then stored in microtubes at −20 °C until analysis was performed. Serum levels of the total protein, albumin, urea, C-reactive protein, cholesterol, fructosamine, alanine aminotransferase (ALT), aspartate aminotransferase (AST), and gamma-glutamyltranspeptidase (GGT) were determined. For this purpose, Analisa^®^ kits (Gold Analisa Diagnóstica Ltda, Belo Horizonte, Brazil) were used with EQUIPIVET equipment (Zybio EXC-200^®^, Shenzhen, China). The globulin concentration was calculated using the following equation: total protein—albumin.

#### 2.5.4. Proteinogram and Cytokines

Firstly, the total protein content obtained using the biuret technique was used as a reference for quantification. Sodium dodecyl sulfate–polyacrylamide gel electrophoresis (SDS-PAGE) was performed according to the technique described by Fagliari et al. [13] using mini-gels (10 × 10 cm) with adaptations made by Tomasi et al. [14]. The gels were stained with Coomassie blue and photographed to identify and quantify the protein fractions using Labimage 1D software (Loccus Biotechnology, São Paulo, Brazil). A standard containing fractions with molecular weights between 10 kDa and 250 KD (Kaleidoscope, Bio-Rad, Hercules, CA, USA) was used as a reference, using known commercial standards. Analyses were performed in duplicate.

Interleukin 1β (IL-1β), IL-6, and IL-10 levels in serum were measured using commercial kits and an enzyme-linked immunosorbent assay device (Awareness Technology, Inc., Palm City, FL, USA; Chem Well; USCN, Wuhan, China). Tumor necrosis factor alpha (TNF-α) in serum was assessed by the ELISA assay using commercial Quantikine immunoassay kits (Abingdon Science Park, Abingdon, UK) according to the manufacturer’s instructions.

#### 2.5.5. Oxidative Status

The oxidative status variables evaluated in the blood serum were reactive oxygen species (ROS), thiobarbituric acid-reactive substances (TBARSs), and myeloperoxidase activity (MPO). The analyses were performed in triplicate using specific biochemical methodologies. Color and fluorometric readers were prepared using Varioskan™ LUX (Thermo Scientific™, Waltham, MA, USA).

ROS formation was estimated using a fluorometric protocol established by Ali et al. [15]. Ten microliters of serum were soaked in 10 µL of 2′,7′-dichlorofluorescein diacetate (DCFH-DA, 7 μM) and 240 µL of PBS. After 30 min of incubation at 37 °C, the final product of DCFH-DA oxidation, dichlorofluorescein (DCF), was measured. The fluorescence emission intensity was read at an emission wavelength of 525 nm and an excitation wavelength of 488 nm. The results were expressed as a percentage (%) of the fluorescence intensity relative to the control.

Lipoperoxidation is a highly rapid reaction formed by the breakdown of polyunsaturated fatty acids, which are usually measured by their products, mainly TBARSs, among which malondialdehyde (MDA) is the primary product [16]. To evaluate this product, the reaction of thiobarbituric acid (TBA) with serum samples was used, which, in the presence of MDA, resulted in a pink product that could be read at 532 nm. Briefly, 20 µL of each sample was mixed with 55 µL distilled water, 100 µL orthophosphoric acid (0.2 M), and 25 µL TBA (0.1 M). Spectrophotometric readings were taken after 45 min of incubation at 37 °C. The results are expressed in nanomolar MDA/mL.

MPO is a heme enzyme produced by inflammatory mediators and released from leukocytes at the injury site. Therefore, MPO levels reflect the activation of neutrophils and lymphocytes. MPO catalyzes the reaction of chloride ions with H_2_O_2_ to generate large amounts of hypochlorous acid (HOCl), which further reacts to generate singlet oxygen and hydroxyl radicals. In the presence of H_2_O_2_ as an oxidizing agent, MPO catalyzes the oxidative coupling of phenol and 4-aminoantipyrine (AAP), resulting in a colored product, quinoneimine, with a maximum absorbance of 492 nm [17]. MPO activity was analyzed using a modified peroxidase system, with a mixture of 12 µL of serum sample with 148 µL of AAP in phenol solution (AAP 2.5 mM; phenol 20 mM) and 17 µL of H_2_O_2_ solution (17 mM). After 30 min of incubation at 37 °C, the system was analyzed spectrophotometrically. The results are expressed as μM quinoneimine per mg of protein produced in 30 min (μMq/mg/30 min).

In whole blood collected with sodium citrate, the activity of antioxidant enzymes, catalase (CAT) and superoxide dismutase (SOD), was measured. Prior to enzyme analysis, protein levels were determined by the method of Bradford [18] using bovine serum albumin as a standard. CAT activity was measured with the modified method of Nelson and Kiesow [19]. This assay involved a change in absorbance at 240 nm due to the CAT-dependent decomposition of hydrogen peroxide. CAT activity was expressed as nmol CAT per milligram protein. SOD activity was determined based on the inhibition of the radical superoxide reaction with adrenalin as described by McCord and Fridovich [20]. In this method, SOD present in the sample competes with the detection system for the superoxide radical. The results were expressed as IU SOD per milligram protein.

### 2.6. Milk Quality: Centesimal Composition, Urea, and Somatic Cell Count of Milk

Analyses of chemical composition, a standard plate count of bacteria, and a somatic cell count (SCC) were performed by the Commercial Milk Quality Laboratory, Curitiba, PR, accredited to the Ministry of Agriculture, Livestock, and Supply by MAPA ordinance. The quantification of fat, protein, lactose, total solids, and the defatted dry extract was performed using the Mid-Infrared Spectrometry Method, according to ISO 9622—IDF Standard 141:2013. The total bacterial count was performed using the Flow Cytometry Method, according to ISO 21187—IDF Standard 196/2021, and the SCC by the Flow Cytometry Method, according to ISO 13366-2—IDF Standard 148-2/2006.

### 2.7. Fatty Acid (FA) Profile in Milk

The extraction was performed using the method described by Bligh and Dyer [21]. Samples (3 mL) were added to 50 mL polypropylene tubes, and 16 mL of methanol and 8 mL of chloroform were added for the homogenization and complete disintegration of the sample, followed by mechanical stirring for 60 min. Thereafter, 8 mL of chloroform and Na_2_SO_4_ 1.5% solution were added to promote a biphasic system. This mixture was shaken for 2 min and then centrifuged for 15 min at 2000 rpm. The lipids obtained from the chloroform phase were subjected to fatty acid analysis.

FA methylation was performed using the transesterification method proposed by Hartman and Lago [8,22]. The extracted lipids were added to 1 mL of 0.4 M KOH methanolic solution in a test tube and shaken in a vortex for 1 min. The samples were kept in a water bath for 10 min at boiling point. Subsequently, they were cooled to room temperature, and 3 mL of 1 M H_2_SO_4_ methanolic solution was added, shaken in a vortex, and maintained in a water bath for 10 min. After cooling, 2 mL hexane was added, and the mixture was centrifuged at 2000 rpm for 10 min. Lastly, hexane with fatty acid methyl esters (FAMEs) was subjected to chromatographic analysis.

For FAME determination, a gas chromatograph (TRACE 1310) equipped with a flame ionization detector (Thermo Scientific) was used. One microliter of sample was injected into a split/splitless injector operated in a 1:10 split mode at 250 °C. Hydrogen was used as the carrier gas at a constant flow rate of 1.5 mL/min. FAME separation was performed using an RT 2560 (100 m × 0.25 mm × 0.20 μm of thickness film, Restek, Bellefonte, PA, USA) chromatography column. The initial oven temperature was programmed to be 100 °C for 5 min and then increased to 180 °C at 8 °C/min. Then, it was increased to 210 °C at 4 °C/min, and finally, until 240 °C, it was increased to 20 °C/min, and it was maintained for 20 min under isothermal conditions. The detector temperature was maintained at 250 °C. The FAME compounds were identified by comparing the experimental retention times to those of authentic standards (FAME Mix-37, Sigma Aldrich, St. Louis, MO, USA). The results were presented as a percentage of each FA identified in the lipid fraction, considering the chain size equivalent factor of FAMEs for FID and the conversion factor of the ester to the respective acid, according to Visentainer and Franco [23].

### 2.8. Statistical Analysis

First, a descriptive analysis of the data was performed by applying the normality test (Shapiro–Wilk) and evaluating the residual error. The data without a normal distribution were transformed to logarithms and normalized for subsequent analyses. All data were analyzed using the SAS ‘MIXED procedure’ (SAS Inst. Inc., Cary, NC, USA; version 9.4) with Satterthwaite’s approximation to determine the denominator degrees of freedom for the fixed-effects test. Mean milk production and feed efficiency variables were tested for a fixed effect of treatment using animals (treatment) as a random effect. The remaining data were analyzed as repeated measures and tested for fixed treatment effects using day and treatment × day, with cow (treatment) as a random effect. The d1 results were included as independent covariates. Additionally, for these variables, the d1 scores were removed from the dataset to generate the mean per treatment but were retained as covariates. The first-order autoregressive covariance structure was selected according to the lowest Akaike information criterion. Means were separated using the PDIFF method (Student’s test), and all results were reported as LSMEANS followed by standard error (SEM). Significance was defined as *p* ≤ 0.05, and a trend was indicated when *p* > 0.05 and ≤ 0.10.

## 3. Results

### 3.1. Productive Performance

The performance results are presented in Table 2 and Figure 1. There was no effect of treatment on the average milk production per cow per day during the adaptation period (d1–14) and experimental period (d15–45) (Table 2). However, we observed a treatment × day interaction (Figure 1): the cows that consumed the phytobiotic additive produced a greater quantity of milk (d14, d17, d18, d30, d39 and d45). We observed 8.57% greater corrected milk production for fat percentage in the cows that consumed phytobiotics than in the control during the experimental period. The effect of the treatment on feed intake was 10.23% lower in cows that consumed the additive than in the control. The feed efficiency values were 15.38% higher in cows that consumed phytobiotics in both calculations using milk production per day and milk production corrected for 4% fat.

### 3.2. Milk Quality

The results of centesimal composition and SCC in milk are presented in Table 3. There was an effect of treatment and day; thus, there was a treatment × day interaction for milk fat, with cows in the phytobiotic group having the highest milk fat compared to the control group. There was no effect of day, treatment, or treatment × day interaction on protein, lactose, total solids, urea, and SCC in milk.

The results of the milk fatty acid profile are presented in Table 4. No effect of treatment was observed for specific fatty acids. Still, there was a tendency for a higher sum of unsaturated fatty acids (*p* = 0.09) and monounsaturated fatty acids (*p* = 0.07) in the milk of cows that consumed the phytogenic. However, there was a small difference for the sum of polyunsaturated fatty acids (control: 1.51 and phytogenic: 1.61%), but this was significant between the groups due to the consumption of the additive. Consequently, we verify that a higher UFA/SFA ratio was observed in the milk of cows that consumed the phytogenic compared to the control.

### 3.3. Hematologic and Biochemical Changes

**Figure 2:**
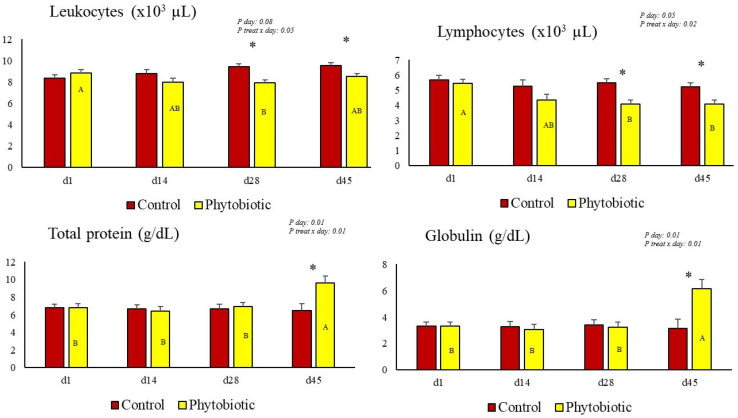
The effect of the treatment vs. day interaction (illustrated by asterisks above the bars) and day effect (shown by capital letters (A and B) inside the phytobiotic group bar) for total leukocyte, lymphocyte, total protein, and globulin biomarkers. The asterisk (*) illustrates the difference on each day, referring to the treatment × day interaction.

The results for blood variables are presented in Table 5 and Figure 2. The effect of the day and treatment × day interaction was observed for total leukocyte and lymphocyte counts, both of which were lower in the blood of cows in the phytobiotic group. There was no effect of treatment or interaction on the other blood count variables (the number of granulocytes, monocytes, platelets, hemoglobin concentration, and hematocrit percentage), except for the number of erythrocytes, which was higher in cows that consumed the additive. Figure 2 illustrates the treatment × day interaction and the effect of the day on leukocyte and lymphocyte counts.

Total protein and globulin levels were higher in the serum of cows in the phytobiotic group compared to the control (Table 5), emphasizing the higher protein concentration on day 45 of the experiment (Figure 2). No effect of treatment or treatment × day interaction was observed for albumin, cholesterol, fructosamine, GGT, AST, ALT, triglycerides, glucose, or urea.

**Figure 3:**
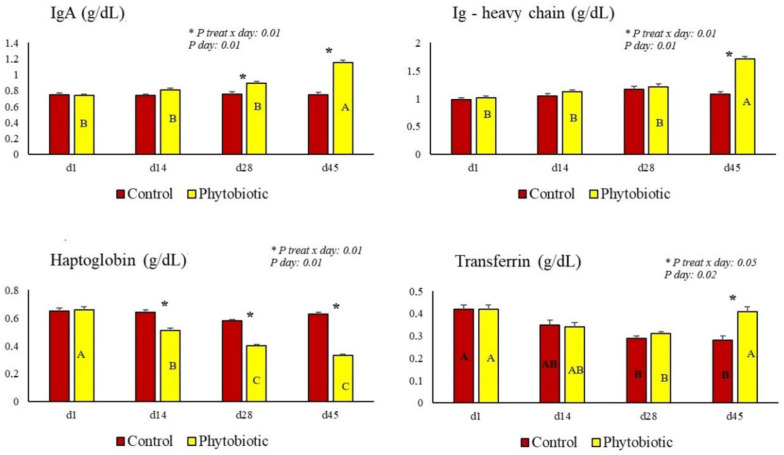
The effect of treatment vs. day interaction (illustrated by asterisks above the bars) and day effect (shown by capital letters (A, B, and C) inside the bar) for biomarkers IgA, Ig-hc, haptoglobin, and transferrin. The asterisk (*) illustrates the difference on each day, referring to the treatment × day interaction.

Protein profiles obtained by electrophoresis are shown in Table 6 and Figure 3. Higher levels of IgA, Ig heavy chain (Ig-hc), and transferrin were observed in the serum of cows fed the phytobiotic (*p* < 0.05). Ceruloplasmin and haptoglobin levels were lower in the serum of these cows than in that of the control (*p* < 0.05). A treatment × day interaction was observed mainly on day 45 for IgA, Ig-hc, haptoglobin, and transferrin (Figure 3).

**Figure 4:**
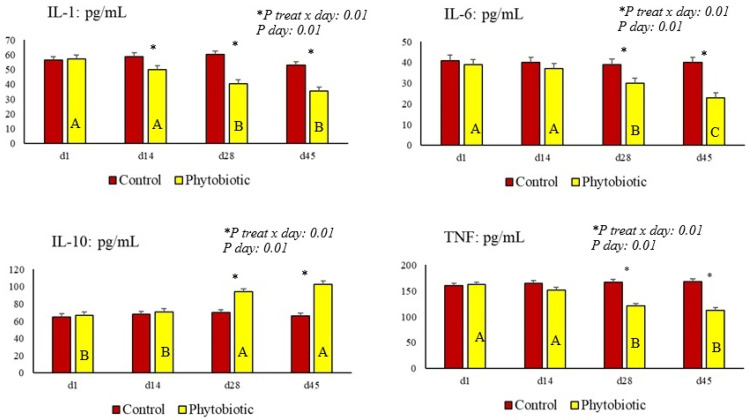
The effect of treatment vs. day interaction (illustrated by asterisks above the bars) and day effect (shown by capital letters (A, B and C) inside the bar) for the cytokine biomarkers (IL-1, IL-6, IL-10 and TNF). The asterisk (*) illustrates the difference on each day, referring to the treatment × day interaction.

We verified the effect of the treatment and day and the treatment x day interaction for cytokine levels (Table 6 and Figure 4). Lower levels of TNF, IL-1 and IL-6 and higher levels of IL-10 in the serum of cows that consumed the phytobiotic compared to the control were observed.

**Figure 5:**
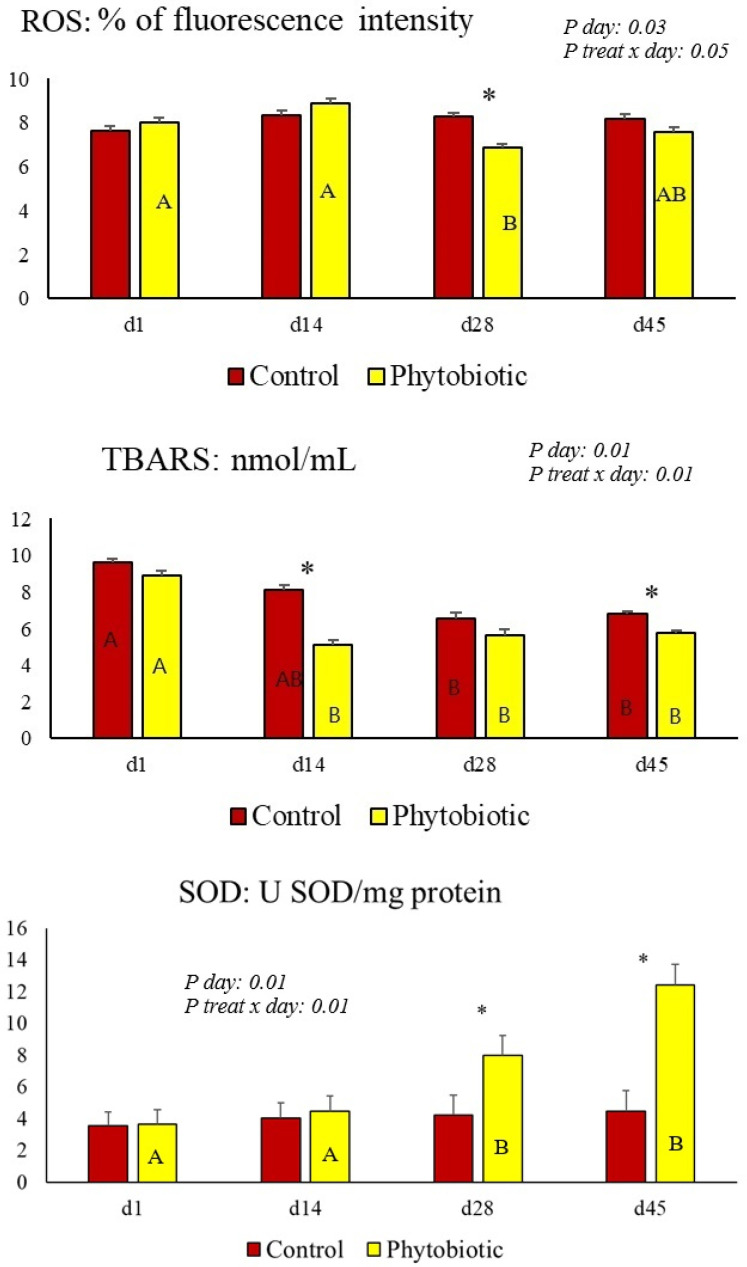
The treatment × day interaction (illustrated by asterisks above the bars) and day effect (shown by capital letters (A and B) inside the bar) for reactive oxygen species (ROS) and lipid peroxidation (TBARS) in serum and superoxide dismutase (SOD) in total blood from phytobiotic-fed cows. The asterisk (*) illustrates the difference on each day, referring to the treatment × day interaction.

Oxidative status markers were presented in Table 6 and Figure 5. There was no treatment effect for ROS, but there was a day effect and treatment × day interaction for this variable, with lower ROS levels observed in the cows in the phytobiotic group compared to the control on day 28 of the experiment. There was a treatment, day, and interaction effect for TBARS levels, which was lower in cows that consumed the phytobiotic on days 14, 28, and 45. MPO activity only had a treatment effect, with higher enzymatic activity in the cows in the phytobiotic group compared to the control. CAT activity was higher in the blood of cows that consumed the phytogenic compared to the control. SOD activity was higher in the blood of cows that consumed additives on days 28 and 45 compared to the control.

## 4. Discussion

In a practical and applied way, these studies showed that the intake of phytobiotic additives by Jersey cows influence productive performance corrected to fat, feed intake, feed efficiency, and fat percentage in milk. Some of these results corroborate those of another recently published study on the photoactivities of Jersey cows [24] and Lacaune sheep [25]; but it is important to emphasize that the studies used different formulations, so a comparison needs to be made with caution. The present study tested cinnamon and oregano EOs in free and microencapsulated forms, unlike the study by Vitt et al. [24], which used the same concentration of these EO only in microencapsulated form. Maxin et al. [26] reported that the fat content in cow’s milk is altered by acetate and butyrate; that is, when they increase, there is a decrease in propionate, glucose, and fat content in milk, which has already been described in other studies with phytobiotics similar to the one tested here [8,9,24]. A lower feed intake may be related to the free EO in the additive, which generated a greater odor in the concentrate and total diet, and reduced intake. Despite this, milk production in these cows was equal between groups; consequently, because when we calculated the feed efficiency value, we found that it was significantly higher in the cows that consumed the phytogenic. Among the additive ingredients tested, we found that turmeric extract contains curcumin, already described as responsible for improving the digestibility of sheep fiber and thus increasing milk production [27], as well as improving the digestibility of feed and nutrients in dairy cows fed *Curcuma longa* extract in the diet [28]. However, we cannot rule out other nutritional mechanisms that are capable of altering milk composition and growth performance, such as how the use of essential oils (oregano and cinnamon) [5] and tannins [29] alters the acetate/propionate ratio and amount of short-chain fatty acids (SCFAs). We observed a day effect for milk fat, which increased throughout the experiment; however, this increase was higher in the phytobiotic group. The explanation for the day effect in both groups is related to the feeding of these cows before starting the experiment, i.e., the cows had access to pasture and were animals in the early phase of lactation (less than 30 days postpartum); this is a physiological and common moment in which the animal is in a negative energy balance and has an unstable metabolism. At the end of the experiment, the percentage of milk fat reached the reference value for the Jersey breed at peak production. The exact mechanism that led to the increase in fat in the animals that consumed the phytobiotic will be investigated in the future by our research group, evaluating ruminal fermentation, volatile fatty acids, and ruminal and intestinal microbiota.

The exact mechanism involved in this increase in milk fat is unknown. However, in the future, we will investigate the influence of this additive on lipid metabolism, which did not change serum cholesterol levels. The additive positively influenced the composition of fatty acids in milk, increasing the index of polyunsaturated fatty acids. The addition of curcumin to dairy sheep feed increases monounsaturated fatty acids [27] and the use of tannins in goats increases oleic acid [30]. According to the literature, tannins can inhibit the last step of ruminal biohydrogenation; therefore, this increases the content of unsaturated fatty acids in milk [31]. However, it is worth noting that the effect of the additive on milk quality was moderate, i.e., only affecting fat. We did not hypothesize that the consumption of the additive would alter protein and lactose levels in the milk; but we expected a reduction in SCC, as had already been described in another study with a phytogenic [24]. The blood results discussed below suggest an anti-inflammatory effect of the additive, which could have reduced the somatic cell count in the milk, but this did not occur, which may be related to other local factors in the mammary gland, which are not known.

Lower blood leukocyte counts due to reduced lymphocytes were observed in cows fed phytobiotics. This change suggests that the additive interferes with the modulation of inflammatory processes [32,33], thus favoring an anti-inflammatory effect. Lower leukocyte counts have already been verified in the consumption of curcumin by dairy sheep [27] and dairy calves [34], as well as the ingestion of microencapsulated cinnamon and oregano oil in the diet of lambs [35]. Adding chestnut tannin to the diet of cows increased leukocyte and lymphocyte counts but reduced the neutrophil/lymphocyte ratio [36]. It is important to make it clear to the reader that the lower leukocyte/lymphocyte count could also indicate immunosuppression, but due to the promising productive results, as well as other blood biomarkers, we believe that this reduction in the count of this cell is an anti-inflammatory effect, confirmed by the lower concentration of pro-inflammatory cytokines (IL-1β, IL-6, and TNF) and higher anti-inflammatory cytokine levels (IL-10).

A higher concentration of total proteins due to increased globulins was observed in this study. As the animals were apparently healthy, without any sign of dehydration, we interpreted the reduction in total protein as an immunological effect, confirmed by the protein profile determined by electrophoresis. Higher globulin levels have already been recorded in lactating goats fed a diet based on cinnamon, ginger, and garlic oils [37]. The same commercial product tested here did not alter total protein and globulin levels when used in the diet of feedlot beef cattle [8]. However, similar additives in a phytogenic blend increased globulin levels in growing steers [9] and lactating cows [24]. A leukogram showed a lower total lymphocyte count. However, the number of globulins was higher, which suggests that there was a reduction in T lymphocytes and possibly an increase in B lymphocytes or more significant activity of these cells, which resulted in a greater production of immunoglobulins, as observed in our study, where cows fed phytobiotics had a higher concentration of serum IgA, similar to that found in other studies by our research group [9,24,38,39]. However, this is the hypothesis of modulation for greater IgA; but we cannot rule out the possibility that the additive causes irritation in the intestinal mucosa, which could also stimulate the increase in this immunoglobulin. It is essential to highlight that the concentration of other heavy-chain immunoglobulins also increases, reinforcing that the ingestion of this additive stimulates a humoral response [40]. There is a hypothesis that immunoglobulins increase by the direct action of the additive’s ingredients on humoral immune cells, and another possibility is that the components of the additive (mainly essential oil and tannin) sensitize the intestinal mucosa, which is an immune and defense response, and increase IgA levels.

The reduction in lymphocytes, combined with a lower concentration of positive acute-phase proteins (ceruloplasmin and haptoglobin) and an increase in harmful acute-phase protein (transferrin), helps to characterize an anti-inflammatory response that has been commonly described in ruminants fed with phytobiotics [24,39,41,42]. It is already known that the combination of microencapsulated oils of carvacrol, cinnamaldehyde, and thymol has anti-inflammatory effects [41,42]. Tung et al. [43] observed that Cinnamomum osmophloeum EO could strongly inhibit nitric oxide (NO) production in RAW 264.7 cells after exposure to lipopolysaccharide (LPS), highlighting the involvement of cinnamaldehyde in this activity. The ability of cinnamaldehyde to reduce inducible nitric oxide synthase (iNOS) mRNA synthesis in RAW 264.7 cells has been noted [44]. Curcumin reduces the activity of enzymes that mediate inflammation, such as cyclooxygenase-2 (COX-2), lipoxygenase (LOX), and iNOS, and reduces the levels of pro-inflammatory cytokines [45,46]. Wijesinghe et al. [47] suggested that plant tannins exert anti-inflammatory effects by inhibiting NO and prostaglandin-E2. In summary, the anti-inflammatory effect observed here was expected and may indirectly be responsible for the higher milk production of these cows because reducing the inflammatory response required less ATP consumption to maintain inflammation, and more energy was available for milk production.

Lower levels of TBARS and ROS combined with the greater activity of antioxidant enzymes CAT and SOD were observed in this study, similar to those described in other studies on phytobiotics in ruminants [8,9,24,39]. It is well known that curcumin has antioxidant effects in sheep [48,49], and cinnamon and oregano EOs decrease lipid peroxidation as they have compounds with a high capacity to absorb oxygen radicals [50] and tannins, which are plant metabolites that have antioxidant activity and can be used to improve the antioxidant status of animals [51]. According to the literature, curcumin has active groups capable of neutralizing free radicals and stimulating antioxidant enzymes such as SOD, glutathione peroxidase, and glutathione S-transferase [52,53]. Furthermore, a meta-analysis showed that curcumin, when consumed, reduces the concentration of malondialdehyde (MDA) in serum and has the potential to increase the total antioxidant potential, consequently removing reactive oxygen and nitrogen, chelating metals, and regulating numerous enzymes [54].

## 5. Conclusions

The inclusion of a phytobiotic additive based on a combination of oregano and cinnamon EO (free and microencapsulated) associated with turmeric and tannin extract in the diet of Jersey cows has positive effects under these experimental conditions, enhancing fat-corrected milk (4%FCM), and it is also capable of increasing fat in milk, generating a partial improvement in milk quality since milk protein, lactose and SCC are not altered. However, the addition of the phytogenic reduced the cows’ feed intake but maintained production, leaving the calculated feed efficiency value of these cows higher. The intake of the additive by the cows had an anti-inflammatory effect, capable of reducing pro-inflammatory markers, such as acute-phase proteins, but stimulated the production of immunoglobulins. A reduction in lipid peroxidation and ROS levels indicates that the additive has an antioxidant effect with higher CAT and SOD activity and can reduce and minimize physiological oxidative stress in lactating cows.

## Figures and Tables

**Table 1:** Chemical composition and fatty acid content of partial mixed ration (PMR) used in cow feed.

Variable	PMR Control ^1^	PMR Phytobiotic ^1^	Robot Feed
Dry matter (DM), %	49.7	49.6	92.0
Ash, % DM	7.24	7.11	10.6
Crude protein, % DM	14.0	14.0	21.8
Ethereal extract, % DM	1.21	1.02	5.63
NDF, % DM	19.0	19.2	29.7
**Fatty acid, %**			
C4:0 (Butyric)	0.55	0.57	-
C6:0 (Caproic)	0.01	1.31	-
C12:0 (Lauric)	0.72	0.70	-
C13:0 (Tridecanoic)	0.20	0.15	-
C14:0 (Myristic)	0.03	0.27	-
C14:1 (Myristoleic)	0.40	0.49	-
C15:0 (Pentadecanoic)	0.16	0.24	-
C16:0 (Palmitic)	23.6	24.1	-
C16:1 (Palmitoleic)	2.15	2.08	-
C17:0 (Heptadecanoic)	0.22	0.47	-
C17:1 (cis-10-Heptadecenoic)	0.04	0.07	-
C18:0 (Stearic)	4.22	4.85	-
C18:1n9t (Elaidic)	0.34	0.33	-
C18:1n9c (Oleic)	24.6	24.2	-
C18:2n6c (Linoleic)	39.6	38.8	-
C20:0 (Arachidic)	0.68	0.53	-
C20:1n9 (cis-11-Eicosenoic)	0.25	0.24	-
C18:3n3 (a-Linolenic)	4.20	4.55	-
C21:0 (Henicosanoic)	0.05	0.03	-
C20:2 (cis-11,14-Eicosadienoic)	0.06	0.03	-
C22:0 (Behenic)	0.61	0.43	-
C20:3n6 (cis-8,11,14-Eicosatrienoic)	0.01	0.02	-
C20:4n6 (Arachidonic)	0.17	0.11	-
C24:0 (Lignoceric)	0.70	0.47	-
C24:1n9 (Nervonic)	0.02	0.07	-

The concentrate used in this diet was formulated based on 34% corn, 42% soybean meal, 5% wheat bran, 12.5% soybean hulls, 1.5% sodium bicarbonate, 4.5% minerals/vitamin supplement, 0.25% urea, and 0.25% mycotoxin adsorbent. The vitamin and mineral supplement had the following guaranteed levels: calcium (min.) 231.40 (g/kg), calcium (max.) 250.00 (g/kg), phosphorus (min.) 40.00 (g/kg), sulfur (min.) 20 (g/kg), magnesium (min.) 25.00 (g/kg), potassium (min.) 10.00 (g/kg), sodium (min.) 70.00 (g/kg), cobalt (min.) 15.00 (mg/kg), copper (min.) 750.00 (mg/kg), copper (min.) 20.00 (mg, kg), iodine (min.) 40.00 (mg/kg), manganese (min.) 2000.00 (mg/kg), selenium (min.) 22.00 (mg/kg), zinc (min.) 2850.00 (mg/kg), vitamin A (min.) 350,000.00 (IU/kg), vitamin D3 (min.) 100,000.00 (IU/kg), vitamin E (min.) 2000.00 (IU/kg). ^1^ Partial mixed ration, with only the diet provided in individual feeders/automatic feeders (without robot feed).

**Table 2:** Milk production (MP), fat-corrected milk (4% FCM).

Variables	Control	Phytobiotic	SEM	*p*: Treat ^2^
Milk production—MP. kg (mean)				
d −7 to 1 (pre-experiment)	22.2	22.6	0.22	0.95
d 1 to 14	22.6	22.8	0.20	0.94
d 15 to 45	25.2	26.0	0.19	0.15
Fat-corrected milk (4%FCM) ^1^. kg				
d −7 to 1	18.5	19.5	0.2	0.84
d 1 to 14	20.2	21.7	0.18	0.76
d 15 to 45	24.5 ^b^	26.6 ^a^	0.17	0.01
Feed intake—FI. kg (mean)				
d 1 to 14	16.5	16.2	0.14	0.95
d 15 to 45	17.6 ^a^	15.8 ^b^	0.12	0.01
Feed efficiency (kg DM/kg FI)				
d 15 to 45	1.43 ^b^	1.65 ^a^	0.04	0.01
Body score				
d1	3.25	3.08	0.08	0.62
d45	3.34	3.11	0.07	0.12

Note ^1^: Milk production corrected for 4% fat (4%FCM) was estimated by the equation proposed by the NRC (2001) [10]: FCM = 0.4 × (kg of milk produced) + 0.15 × (% fat) × (kg of milk produced). Note ^2^: The treatment effect when *p* < 0.05, with the difference between groups illustrated by different lowercase letters (a and b) in the same row.

**Table 3:** Centesimal composition of milk from cows that consumed phytobiotics, milk urea levels, and somatic cell count (SCC).

Variables	Control	Phytobiotic	SEM	*p*: Treat ^1^	*p*: Day ^2^	*p*: Treat × Day ^3^
Fat (g/kg)	36.5 ^b^	40.1 ^a^	0.47	0.01	0.05	0.01
Protein (g/kg)	36.9	36.8	0.35	0.97	0.96	0.97
Lactose (g/kg)	46.3	45.8	0.24	0.88	0.95	0.93
Total solids (g/kg)	120	123	1.75	0.91	0.96	0.92
Urea (mg/dL)	18.3	19.9	0.64	0.82	0.89	0.83
SCC (×10^3^ mL)	61.9	56.3	2.42	0.64	0.58	0.72
Fat (g/kg) ^2,3^						
d14	32.9 ^bB^	36.9 ^aB^	0.46			
d28	34.1 ^bAB^	37.8 ^aB^	0.48			
d45	42.4 ^bA^	45.6 ^aA^	0.46			

Note ^1^: The treatment effect when *p* < 0.05, with the difference between groups illustrated by different lowercase letters (a and b) in the same row. Note ^2^: The day effect when *p* < 0.05, illustrated by uppercase letters (A, and B) in the same column for milk fat in both groups. Note ^3^: This table demonstrates the treatment versus day interaction for fat on days 14, 28, and 45 of the experiment, with lowercase letters (a and b) in the same row.

**Table 4:** Fatty acid profile in milk from cows that consumed phytobiotics as feed additive.

Fatty Acid	Control	Phytobiotic	SEM	*p*-Value ^1^
C4:0 (Butyric)	0.20	0.19	0.016	0.95
C6:0 (Caproic)	0.09	0.01	0.033	0.24
C10:0 (Capric)	2.42	2.10	0.156	0.36
C11:0 (Undecanoic)	0.21	0.15	0.024	0.23
C12:0 (Lauric)	3.20	3.11	0.078	0.78
C13:0 (Tridecanoic)	0.13	0.12	0.005	0.96
C14:0 (Myristic)	12.0	12.5	0.158	0.92
C14:1 (Myristoleic)	0.53	0.53	0.032	0.94
C15:0 (Pentadecanoic)	1.11	1.24	0.044	0.56
C16:0 (Palmitic)	43.9	43.1	0.504	0.91
C16:1 (Palmitoleic)	1.17	1.06	0.043	0.44
C17:0 (Heptadecanoic)	0.66	0.67	0.009	0.98
C17:1 (cis-10-Heptadecenoic)	0.12	0.11	0.004	0.95
C18:0 (Stearic)	10.8	11.9	0.275	0.18
C18:1n9t (Elaidic)	1.50	1.71	0.058	0.13
C18:1n9c (Oleic)	14.4	15.0	0.369	0.31
C18:2n6t (Linolelaidic)	0.02	0.01	0.001	0.99
C18:2n6c (Linoleic)	1.25	1.33	0.027	0.19
C20:0 (Arachidic)	0.14	0.16	0.003	0.35
C20:1n9 (cis-11-Eicosenoic)	0.07	0.08	0.003	0.96
C18:3n3 (a-Linolenic)	0.10	0.12	0.006	0.55
C21:0 (Henicosanoic)	0.20	0.25	0.016	0.47
C20:2 (cis-11.14-Eicosadienoic)	0.02	0.01	0.002	0.96
C22:0 (Behenic)	0.06	0.07	0.002	0.95
C20:3n6 (cis-8.11.14-Eicosatrienoic)	0.07	0.07	0.002	0.99
C20:4n6 (Arachidonic)	0.04	0.04	0.002	0.98
C22:2 (cis-13.16-Docosadienoic)	0.02	0.01	0.003	0.96
C24:0 (Lignoceric)	0.05	0.04	0.005	0.94
C20:5n3 (cis-5.8.11.14.17-Eicosapentaenoic)	0.01	0.01	0.000	0.99
C24:1n9 (Nervonic)	0.01	0.01	0.001	0.99
SFA ^2^	75.3	74.7	0.652	0.24
UFA	19.3	20.1	0.125	0.09
MUFA	17.8	18.5	0.097	0.07
PUFA	1.51 ^b^	1.61 ^a^	0.030	0.03
UFA/SFA	0.25 ^b^	0.27 ^a^	0.011	0.05

Note ^1^: Different letters in the same row illustrate the treatment effect when *p* ≤ 0.05. Note ^2^. saturated fatty acids (SFA), unsaturated fatty acids (UFA), monounsaturated fatty acids (MUFA) and polyunsaturated fatty acids (PUFA).

**Table 5:** Hemogram and serum biochemistry of cows fed phytobiotics.

Variables	Control	Phytobiotic	SEM	*p*: Treat ^1^	*p*: Day ^2^	*p*: Treat × Day ^2^
Hematology						
Total leukocytes (×10^3^ μL)	9.22 ^a^	8.02 ^b^	0.35	0.05	0.08	0.05
Lymphocytes (×10^3^ μL)	5.33 ^a^	4.18 ^b^	0.23	0.05	0.05	0.02
Granulocytes (×10^3^ μL)	3.16	3.26	0.20	0.89	0.82	0.93
Monocytes (×10^3^ μL)	0.72	0.79	0.04	0.87	0.79	0.81
Erythrocytes (×10^6^ μL)	4.98 ^b^	5.44 ^a^	0.08	0.05	0.18	0.12
Hemoglobin (g/dL)	9.10	9.31	0.14	0.26	0.31	0.26
Hematocrit (%)	26.1	26.4	0.41	0.92	0.94	0.88
Platelets (×10^3^ μL)	370	339	20.9	0.55	0.63	0.61
Serum biochemistry						
Albumin (g/dL)	3.40	3.45	0.08	0.97	0.95	0.92
Total cholesterol (mg/dL)	140	139	1.21	0.91	0.89	0.89
Frutosamine (mg/dL)	325	330	2.05	0.85	0.81	0.87
GGT (U/L) ^3^	30.1	31.0	1.25	0.95	0.94	0.94
Glucose (mg/dL)	64.4	60.7	2.36	0.88	0.84	0.85
Total protein (g/dL)	6.67 ^b^	7.77 ^a^	0.11	0.05	0.01	0.01
AST (U/L) ^3^	88.7	94.6	3.32	0.56	0.26	0.39
ALT (U/L) ^3^	71.3	72.2	2.67	0.92	0.94	0.94
Triglycerides (mg/dL)	10.2	9.95	0.98	0.96	0.95	0.97
Urea (mg/dL)	42.4	45.8	1.54	0.66	0.51	0.45
Globulin (g/dL)	3.26 ^b^	4.31 ^a^	0.13	0.05	0.01	0.01

Note ^1^: Treatment effect when *p* < 0.05, with difference between groups illustrated by different lowercase letters (a, b) in same line. Note ^2^: Day effect and treatment × day interaction when *p* < 0.05; results are shown in Figure 2. Note ^3^: GGT (gamma-glutamyl transferase), AST (aspartate aminotransferase) and ALT (alanine aminotransferase).

**Table 6:** Protein profile in electrophoresis and markers of oxidative reactions of cows fed phytobiotics.

Variables	Control	Phytobiotic	SEM	*p*: Treat ^1^	*p*: Day ^2^	*p*: Treat × Day ^2^
Proteinogram						
IgA (g/dL)	0.75 ^b^	0.98 ^a^	0.03	0.01	0.01	0.01
Heavy-chain Ig (g/dL)	1.12 ^b^	1.46 ^a^	0.05	0.01	0.01	0.01
Ceruloplasmin (g/dL)	0.65 ^a^	0.52 ^b^	0.02	0.05	0.22	0.26
Haptoglobin (g/dL)	0.61 ^a^	0.42 ^b^	0.02	0.01	0.01	0.01
Ferritin (g/dL)	0.40	0.37	0.01	0.65	0.74	0.68
Transferrin (g/dL)	0.32 ^b^	0.36 ^a^	0.01	0.05	0.02	0.05
Cytokines						
IL-1β (pg/mL)	56.9 ^a^	42.9 ^b^	2.58	0.01	0.01	0.01
IL-6 (pg/mL)	40.1 ^a^	29.5 ^b^	2.27	0.04	0.01	0.01
IL-10 (pg/mL)	65.8 ^b^	94.3 ^a^	3.41	0.01	0.01	0.01
TNF (pg/mL)	164 ^a^	135 ^b^	5.02	0.03	0.01	0.01
Oxidative status						
ROS ^3^ (% of fluorescence intensity)	8.20 ^a^	7.60 ^b^	0.22	0.19	0.03	0.05
TBARS (nmol/mL)	6.83 ^a^	5.77 ^b^	0.13	0.02	0.01	0.01
MPO (µM quinoneimin/30 min)	2.27	2.75	0.02	0.05	0.14	0.20
CAT (nmol CAT/mg of protein)	9.74	11.2	0.45	0.04	0.11	0.13
SOD (U SOD/mg of protein)	4.23	8.23	0.31	0.01	0.01	0.01

Note ^1^: The treatment effect when *p* < 0.05, with the difference between groups illustrated by different lowercase letters (a and b) in the same line. Note ^2^: The day effect and treatment × day interaction when *p* < 0.05, with the results presented in Figure 3 and Figure 4. Note ^3^. reactive oxygen species (ROS), thiobarbituric acid reactive substances (TBARS), myeloperoxidase (MPO), catalase (CAT) and superoxide dismutase (SOD).

## Data Availability

The original contributions presented in this study are included in the article. Further inquiries can be directed to the corresponding author.

## References

[1] Van Boeckel T.P., Pires J., Silvester R., Zhao C., Song J., Criscuolo N.G., Laxminarayan R. (2019). Global trends in antimicrobial resistance in animals in low- and middle-income countries. Science.

[2] Patra A.K., Saxena J. (2010). Exploitation of dietary tannins to improve rumen metabolism and ruminant nutrition. J. Sci. Food Agric..

[3] Benchaar C., Calsamiglia S., Chaves A.V., Fraser G.R., Colombatto D., McAllister T.A., Beauchemin K.A. (2008). A review of plant-derived essential oils in ruminant nutrition and production. Anim. Feed Sci. Technol..

[4] Khan H., Ullah H., Nabavi S.M. (2018). Mechanistic insights of hepatoprotective effects of curcumin: Therapeutic updates and future prospects. Food Chem. Toxicol..

[5] Calsamiglia S., Busquet M., Cardozo P., Castillejos L., Ferret A. (2007). Invited review: Essential oils as modifiers of rumen microbial fermentation. J. Dairy Sci..

[6] Yarru L.P., Settivari R.S., Gowda N.K.S., Antoniou E., Ledoux D.R., Rottinghaus G.E. (2009). Effects of turmeric (*Curcuma longa*) on the expression of hepatic genes associated with biotransformation, antioxidant, and immune systems in broiler chicks fed aflatoxin. Poult. Sci..

[7] Vieira L.V., Schmidt A.P., Barbosa A.A., de Oliveira Feijó J., Brauner C.C., Rabassa V.R., Corrêa M.N., Schmitt E., Del Pino F.A.B. (2020). Utilização de taninos como aditivo nutricional na dieta de ruminantes. Arq. De Ciênc. Vet. E Zool. Da UNIPAR.

[8] Lago R.V.P., Wolschick G.J., Signor M.H., Giraldi G.C., Mollosse V.L., Deolindo G.L., Cecere B.G.O., Brunetto A.L.R., Cucco D.C., Bendeti P.D.B. (2024). A mixture of free and microencapsulated essential oils combined with turmeric and tannin in the diet of cattle in the growing and finishing phase: A new tool to enhance productivity. Anim. Feed. Sci. Technol..

[9] Brunetto A.L., Giacomelli C.M., Favero J.F., Bissacotti B.F., Copeti P.M., Morsch V.M., Da Silva A.S. (2023). Phytogenic blend in the diet of growing Holstein steers: Effects on performance, digestibility, rumen volatile fatty acid profile, and immune and antioxidant responses. Anim. Feed. Sci. Technol..

[10] National Research Council (NRC) (2001). Nutrient Requirements of Dairy Cattle.

[11] Silva D.J., Queiroz A.C. (2002). Análises de Alimentos (Métodos Químicos e Biológicos).

[12] Van Soest P.J., Wine R.H. (1968). Determination of lignin and cellulose in acid detergent fiber with permanganate. J. Assoc. Agric. Chem..

[13] Fagliari J.J., Santana A.E., Lucas F.A., Campos E., Curi P.R. (1998). Constituintes Sangüíneos De bovinos recém-nascidos das raças nelore (Bos indicus) e holandesa (Bos taurus) e de bubalinos (Bubalus bubalis) da raça Murrah. Arq. Bras. Med. Vet. Zootec..

[14] Tomasi T., Volpato A., Pereira W.A.B., Debastiani L.H., Bottari N.B., Morsch V.M., Schetinger M.R.C., Leal M.L.R., Machado G., Da Silva A.S. (2018). Metaphylactic effect of minerals on the immune response, biochemical variables and antioxidant status of newborn calves. J. Anim. Physiol. Anim. Nutr..

[15] Ali S.F., LeBel C.P., Bondy S.C. (1992). Reactive oxygen species formation as a biomarker of methylmercury and trimethyltin neurotoxicity. Neurotoxicology.

[16] Jentzsch A.M., Bachmann H., Furst P., Biesalski H.K. (1996). Improved analysis of malondialdehyde in human body fluids. Free. Radic. Biol. Med..

[17] Suzuki K., Ota H., Sasagawa S., Sakatani T., Fujikura T. (1983). Assay method for myeloperoxidase in human polymorphonuclear leukocytes. Anal. Biochem..

[18] Bradford M.M. (1976). A rapid and sensitive method for the quantification of microgram quantities of protein utilizing the principle of protein-dye binding. Anal. Biochem..

[19] Nelson D.P., Kiesow L.A. (1972). Enthalpy of decomposition of hydrogen peroxide by catalase at 25 °C (with molar extinction coefficients of H_2_O_2_ solutions in the UV). Anal. Biochem..

[20] McCord J.M., Fridovich I. (1969). Superoxide dismutase: An enzymatic function for erythrocuprein (hemocuprein). J. Biol. Chem..

[21] Bligh E.G., Dyer W.J. (1959). A rapid method of total lipid extraction and purification. Can. J. Biochem. Physiol..

[22] Hartman L., Lago R.C. (1973). Rapid preparation of fatty acid methyl esters from lipids. Lab. Pract..

[23] Visentainer J.V., Franco M.R.B. (2006). Fatty Acids in Oils and Fats: Identification and Quantification.

[24] Vitt M.G., Signor M.H., Corrêa N.G., Breancini M., Wolschick G.J., Klein B., Silva L.E.L., Wagner R., Jung C.T.K., Kozloski G.V. (2024). Combination of phytoactives in the diet of lactating Jersey cows: Effects on productive efficiency, milk composition and quality, ruminal environment, and animal health. Animals.

[25] Cunha M.G., Alba D.F., Leal K.W., Marcon H., Milarch C.F., Baldissera M.D., Kavalek R.L., Kempka A.P., Vedovatto M., Silva A.S. (2023). Microencapsulated herbal components in the diet of Lacaune ewes: Impacts on physiology and milk production and quality. An. Acad. Bras. Cienc..

[26] Maxin G., Rulquin H., Glasser F. (2011). Response of milk fat concentration and yield to nutrient supply in dairy cows. Animal.

[27] Jaguezeski A.M., Perin G., Bottari N.B., Wagner R., Fagundes M.B., Schetinger M.R.C., Morsch V.M., Stein C.S., Moresco R.N., Barreta D.A. (2018). Addition of curcumin to the diet of dairy sheep improves health, performance and milk quality. Anim. Feed. Sci. Technol..

[28] Hashemzadeh-Cigari F., Khorvash M., Ghorbani G.R., Kadivar M., Riasi A., Zebeli Q. (2014). Effect of supplementation with a phytobiotics-rich herbal mixture on performance, udder health, and metabolic status of Holstein cows with various levels of milk somatic cell counts. J. Dairy Sci..

[29] Wang Z., Zhao Y., Lan X., He J., Wan F., Shen W., Tang S., Zhou C., Tan Z., Yang Y. (2022). Tannic acid supplementation in the diet of Holstein bulls: Impacts on production performance, physiological and immunological characteristics, and ruminal microbiota. Front. Nutr..

[30] Abo-Donia F.M., Yang L.Y., Hristov A.N., Wang M., Tang S.X., Zhou C.S., Han X., Kang J., Tan Z., He Z. (2017). Effects of tannins on the fatty acid profiles of rumen fluids and milk from lactating goats fed a total mixed ration containing rapeseed oil. Livest. Sci..

[31] Vasta V., Makkar H.P.S., Mele M., Priolo A. (2009). Ruminal biohydrogenation as affected by tannins in vitro. Br. J. Nutr..

[32] Bechara G.H., Szabó M.P.J. (2006). Processo Inflamatório. Componente e Eventos Celulares, UNESP. https://www.fcav.unesp.br/Home/departamentos/patologia/GERVASIOHENRIQUEBECHARA/INFLAM_2006.pdf.

[33] Hatcher H., Planalp R., Cho J., Torti F.M., Torti S.V. (2008). Curcumin: From ancient medicine to current clinical trials. Cell. Mol. Life Sci..

[34] Glombowsky P., Volpato A., Campigotto G., Soldá N.M., da S dos-Santo D., Bottari N.B., Schetinger M.R.C., Morsch V.M., Rigon F., Schogor A.L.B. (2020). A adição dietética de curcumina favorece o ganho de peso e possui ação antioxidante, antiinflamatória e anticoccidiana em bezerros leiteiros. Rev. Colomb. Ciênc. Pecu..

[35] Favaretto J.A., Alba D.F., Marchiori M.S., Marcon H.J., Souza C.F., Baldissera M.D., Bianchi A.E., Zanluchi M., Klein B., Wagner R. (2020). Supplementation with a blend based on micro-encapsulated carvacrol, thymol, and cinnamaldehyde in lambs feed inhibits immune cells and improves growth performance. Livest. Sci..

[36] Prodanović R., Nedić S., Vujanac I., Bojkovski J., Nedić S., Jovanović L., Kirovski D., Borozan S. (2023). Dietary supplementation of chestnut tannins in prepartum dairy cows improves antioxidant defense mechanisms interacting with thyroid status. Metabolites.

[37] Kholif A.E., Hassan A.A., El Ashry G.M., Bakr M.H., El-Zaiat H.M., Olafadehan O.A., Matloup O.H., Sallam S.M.A. (2020). Phytogenic feed additives mixture enhances the lactational performance, feed utilization and ruminal fermentation of Friesian cows. Anim. Biotechnol..

[38] Nora L., Marcon C., Deolindo G.L., Signor M.H., Muniz A.L., Bajay M.M., Copetti P.M., Bissacotti B.F., Morsch V.M., da Silva A.S. (2024). The Effects of a Blend of Essential Oils in the Milk of Suckling Calves on Performance, Immune and Antioxidant Systems, and Intestinal Microbiota. Animals.

[39] Leal K.W., Leal M.L.R., Breancini M., Signor M.H., Vitt M.G., Silva L.E.L., Wagner R., Jung C.T.K., Kozloski G.V., de Araujo R.C. (2024). Essential oils and capsaicin in the diet of Jersey cows at early lactation and their positive impact on anti-inflammatory, antioxidant and immunological responses. Trop. Anim. Health Prod..

[40] Tothova C., Nagy O., Kovac G. (2014). Acute phase proteins and their use in the diagnosis of diseases in ruminants: A review. Vet. Med..

[41] Fachini-Queiroz F.C., Kummer R., Estevão-Silva C.F., Carvalho M.D.D.B., Cunha J.M., Grespan R., Bersani-Amado C.A., Cuman R.K.N. (2012). Effects of thymol and carvacrol, constituents of *Thymus vulgaris* L. essential oil, on the inflammatory response. Evid.-Based Complement. Altern. Med..

[42] Gholijani N., Gharagozloo M., Farjadian S., Amirghofran Z. (2016). Modulatory effects of thymol and carvacrol on inflammatory transcription factors in lipopolysaccharide-treated macrophages. J. Immunotoxicol..

[43] Tung Y.-T., Chua M.-T., Wang S.-Y., Chang S.-T. (2008). Anti-inflammation activities of essential oil and its constituents from indigenous cinnamon (*Cinnamomum osmophloeum*) twigs. Bioresour. Technol..

[44] Kim B.H., Lee Y.G., Lee J., Lee J.Y., Cho J.Y. (2010). Regulatory effect of cinnamaldehyde on monocyte/macrophage-mediated inflammatory responses. Mediat. Inflamm..

[45] Menon V.P., Sudheer A.R. (2007). Antioxidant and anti-inflammatory properties of curcumin. Adv. Exp. Med. Biol..

[46] Peng Y., Ao M., Dong B., Jiang Y., Yu L., Chen Z., Hu C., Xu R. (2021). Anti-inflammatory effects of curcumin in the inflammatory diseases: Status, limitations and countermeasures. Drug Des. Dev. Ther..

[47] Wijesinghe W., Ahn G., Lee W.W., Kang M.C., Kim E.A., Jeon Y.J. (2013). Anti-inflammatory activity of phlorotannin-rich fermented Ecklonia cava processing by-product extract in lipopolysaccharide-stimulated RAW 264.7 macrophages. J. Appl. Phycol..

[48] Molosse V.L., Souza C.F., Baldissera M.D., Glombowsky P., Campigotto G., Cazaratto C.J., Stefani L.M., Silva A.S.D. (2019). Diet supplemented with curcumin for nursing lambs improves animal growth, energetic metabolism, and performance of the antioxidant and immune systems. Small Rumin. Res..

[49] Marcon H., Souza C.F., Baldissera M.D., Alba D.F., Favaretto J.A., Santos D.S., Da Silva A.S. (2021). Effect of curcumin dietary supplementation on growth performance, physiology, carcass characteristics and meat quality in lambs. Ann. Anim. Sci..

[50] Bentayeb K., Vera P., Rubio C., Nerin C. (2009). Adaptation of the ORAC assay to the common laboratory equipment and subsequent application to antioxidant plastic films. Anal. Bioanal. Chem..

[51] Soldado D., Bessa R.J.B., Jerónimo E. (2021). Condensed Tannins as Antioxidants in Ruminants-Effectiveness and Action Mechanisms to Improve Animal Antioxidant Status and Oxidative Stability of Products. Animals.

[52] Madrigal-Santillan E., Madrigal-Bujaidar E., Alvarez-Gonzalez I., Sumaya-Martínez M.T., Gutiérrez-Salinas J., Bautista M., Morales-Gonzalez A., García-Luna Y., Gonzalez-Rubio M., Aguilar-Faisal J.L. (2014). Review of natural products with hepatoprotective effects. World J. Gastroenterol..

[53] Wellington K., Jarvis B. (2001). Silymarin: A Review of its Clinical Properties in the Management of Hepatic Disorders. BioDrugs.

[54] Jakubczyk K., Drużga A., Katarzyna J., Skonieczna-Żydecka K. (2020). Antioxidant potential of curcumin—A meta-analysis of randomized clinical trials. Antioxidants.

